# The Combination of the Tunnel View and Weight-Bearing Anteroposterior Radiographs Improves the Detection of Knee Arthritis

**DOI:** 10.1155/2016/9786924

**Published:** 2016-01-26

**Authors:** Oladapo M. Babatunde, Jonathan R. Danoff, David A. Patrick, Jonathan H. Lee, Jonathan K. Kazam, William Macaulay

**Affiliations:** ^1^Center for Hip and Knee Replacement (CHKR), Department of Orthopaedic Surgery, NewYork-Presbyterian Hospital, Columbia University Medical Center, 622 W. 168th Street, PH 1155, New York, NY 10032, USA; ^2^Department of Radiology, NewYork-Presbyterian Hospital, Columbia University Medical Center, 177 Fort Washington Avenue 3-256, New York, NY 10032, USA

## Abstract

Imaging used for the evaluation of knee pain has historically included weight-bearing anteroposterior (AP), lateral, and sunrise radiographs. We wished to evaluate the utility of adding the weight-bearing (WB) posteroanterior (PA) view of the knee in flexion. We hypothesize that (1) the WB tunnel view can detect radiographic osteoarthritis (OA) not visualized on the WB AP, (2) the combination of the AP and tunnel view increases the radiographic detection of OA, and (3) this may provide additional information to the clinician evaluating knee pain. We retrospectively reviewed the WB AP and tunnel view radiographs of 100 knees (74 patients) presenting with knee pain and analyzed for evidence of arthritis. The combination of the WB tunnel view and WB AP significantly increased the detection of joint space narrowing in the lateral (*p* < 0.001) and medial (*p* = 0.006) compartments over the AP view alone. The combined views significantly improved the identification of medial subchondral cysts (*p* = 0.022), sclerosis of the lateral tibial plateau (*p* = 0.041), and moderate-to-large osteophytes in the medial compartment (*p* = 0.012), intercondylar notch (*p* < 0.001), and tibial spine (*p* < 0.001). The WB tunnel view is an effective tool to provide additional information on affected compartments in the painful knee, not provided by the AP image alone.

## 1. Introduction

The orthopedic work-up of knee pain begins with a thorough history and physical examination. Radiographic imaging can then be used to determine the appropriate diagnosis, treatment, and prognosis for the patient. The standard radiographic imaging used for the initial evaluation of knee pain has historically included the weight-bearing (WB) anteroposterior (AP), the lateral, and the sunrise/Merchant view X-rays. At our tertiary care institution and referral center, we often review outside X-rays in consultation, which do not include weight-bearing or flexed knee views. Without these weight-bearing or flexed knee views, there is difficulty in the ability to detect and grade possible radiographic osteoarthritis. Based on these experiences, we became more interested in how the addition of a Rosenberg or tunnel view X-ray would affect the detection, determination, and possible grade of visible radiographic osteoarthritis.

Initially proposed by Holmblad in 1937, the PA view of the knee would provide an increased visualization of both the knee joint space and the intercondylar notch. He described a PA view obtained with the patient kneeling on the radiographic table and the knee in 75° of flexion. With this increased visualization, he stated that more osteophytes, loose bodies, and foreign bodies could be identified using this technique [1]. Since then, several similar techniques, such as the Rosenberg, the Camp-Coventry, the Béclère, and the Schuss, have been described in the literature, all with the objective to further expand the visualization seen with standard AP X-rays.

The Rosenberg method, described in 1988 by Dr. Rosenberg, is a weight-bearing PA radiograph taken with the knee in 45° of flexion [2]. The Rosenberg method was developed to gain insight into the narrowing of cartilage space seen intraoperatively, but not visible on the extension weight-bearing AP radiograph alone [2]. By performing radiographs using this method, increased sensitivity and specificity were seen in comparison to conventional radiographs as flexion allowed greater visibility of cartilage more susceptible to degeneration in the contact zones of the knee [2]. Other methods have been employed such as the Camp-Coventry method (prone position, 40–50° flexion), Béclere (supine position, 60° flexion), and the Schuss view (PA weight-bearing, 30–40° flexion) all to increase visibility of the knee joint space [3, 4]. Ritchie et al. found that when the extension AP radiograph was replaced with the Schuss view, the performance of arthroscopies was reduced by 50% with a move toward definitive surgery to an increased visibility of degenerative changes [4]. Although these methods all apply differing angles of flexion, recent studies have found no consensus on the best flexion angle at which to observe the knee joint space [5, 6].

Multiple studies have previously claimed that there is importance in taking weight-bearing radiographs to determine OA diagnosis in the knee [7–10]. Resnick and Vint utilized this information when producing a trial series of six patients using the Holmblad or “tunnel” view PA approach, which demonstrated an increased observation of destroyed cartilage [11]. The literature is inconclusive with studies demonstrating that a combination of views is optimal for osteoarthritis identification [12–18] and others stating that there is no evidence that there is clinical value to the tunnel view [19, 20]. Similar to the preliminary information produced by Resnick and Vint [11], we believe that the AP view radiograph does not detect all radiographically significant signs of degenerative changes in the knee. In this study, we hypothesize that (1) the WB tunnel view is able to detect radiographic osteoarthritis that the WB AP alone cannot detect, (2) by using both the AP and tunnel view in combination the ability to detect radiographic knee OA is increased, and (3) the added information provided by the tunnel view will assist in evaluation and determination of possible treatment strategies.

## 2. Materials and Methods

After receiving institutional review board approval, we identified patients presenting with knee pain who had been seen at our institution by an adult reconstruction fellowship-trained orthopedic surgeon. Patients were included in the study if both a WB AP and WB tunnel view radiograph were obtained of the affected, painful knee. Patients were excluded from the study if the affected knee had prior surgery. A consecutive cohort of 100 knees (78 patients) were included in the study. Although we recognize that some practitioners utilize the lateral and patellofemoral views to assess the tibiofemoral joint space [21], we believe the AP radiograph is sufficient, and the lateral and sunrise views provide more information regarding the patellofemoral joint. Additionally, the medial and lateral compartments are difficult to differentiate on the lateral view radiograph, which is a critical aspect to this study. The tunnel view at our institution is performed according to the Rosenberg technique. This 45° flexion, posteroanterior, weight-bearing view of the knee is taken with the patella touching the image receptor. The X-ray tube is 40 inches (101.6 cm) away from the image receptor which is centered at the patellae and pointing 10° caudad.

Blinded radiographs were reviewed by two fellowship trained adult reconstruction orthopedic surgeons and one musculoskeletal radiologist. Data collection was performed using an electronic data collection form (eDCF) as follows.

*The Electronic Data Collection Form Used by Investigators*

**Figure 1:**
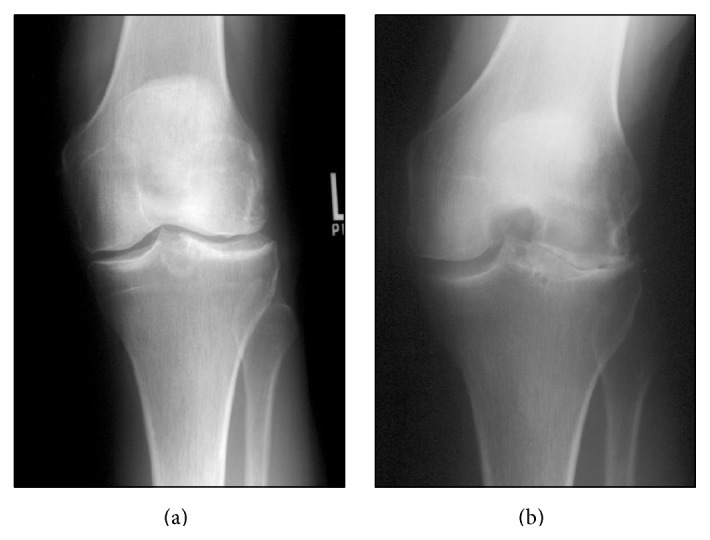
AP radiograph of a left knee (a). The tunnel view of the same knee demonstrates significant degenerative joint disease (b).

**Figure 2:**
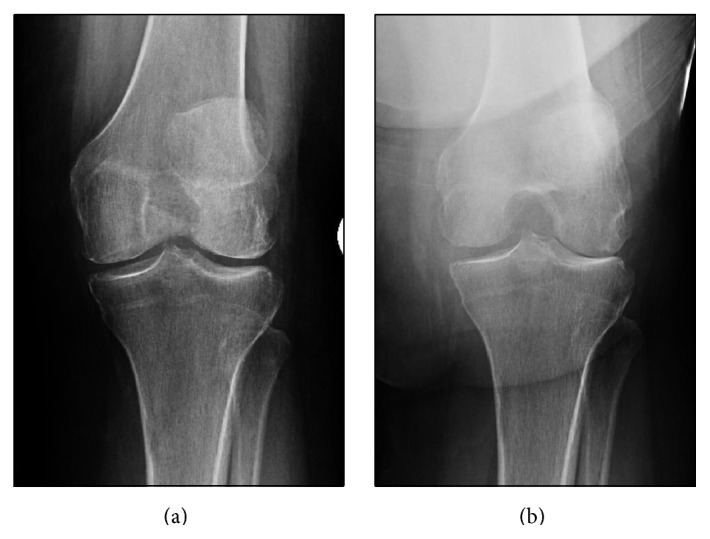
AP radiograph of a left knee (a). The tunnel view shows lateral compartment joint space narrowing (b).

 Investigator Initials:
 Knee #:
 View:
   □ AP
   □ Tunnel
 Compartment:
   □ Medial
   □ Lateral
 Joint Space Narrowing:
   □ None
   □ <25%
   □ 25–49%
   □ 50–75%
   □ >75%
 Sclerosis in:
   □ Tibial plateau
   □ Femoral condyle
 Presence of:
   □ Subchondral cysts
   □ Loose bodies
 Subchondral Tibial Defect:
   □ <5 mm
   □ 5–10 mm
   □ >10 mm
 Subchondral Femoral Defect:
   □ <5 mm
   □ 5–10 mm
   □ >10 mm
 Osteophytes:
   □ None
   □ Small
   □ Moderate
   □ Large
 Intercondylar Notch Osteophytes:
   □ None
   □ Small
   □ Moderate
   □ Large
 Tibial Spine Osteophytes:
   □ None
   □ Small
   □ Moderate
   □ Large

containing quantitative variables for the radiographic criteria of osteoarthritis from both the Kellgren-Lawrence (KL) and the Ahlback scales [7, 22]. Based on arthroscopic correlations by Fife et al., joint space narrowing (JSN) of 50% was determined to be the comparative percentage to indicate a clinically significant difference in joint degeneration [23]. Ordinal values were later assigned for data collection and statistical analysis. Sclerosis, loose bodies, subchondral cysts, subchondral defects, and osteophytes were also evaluated in each view. All variables were independently assessed on the AP and tunnel views. Comparative radiograph images can be seen in Figures 1 and 2.

Prior to initiation of this study, a power analysis was performed and showed that 85 knees per group were required to detect an effect size of 0.5 using our ordinal scale with a power of 90% and significance (*α*) of 0.05. For statistical analysis, the mean of the values assigned by the three physicians was used to create a singular value for both the AP and tunnel views. Each data variable was divided into four distinct categories for analysis: identified in both views, AP only, and tunnel view only and not identified in either view. Using this breakdown, we were able to compare the osteoarthritic changes visible in only the AP view compared to those identified in a radiographic series using both the AP and tunnel views. A *z*-test was used to determine if the tunnel view's addition created a statistically significant change in visible osteoarthritic changes. Statistical analysis was done using IBM SPSS Statistics version 20.0 (IBM SPSS for Windows, rel. 20.0, 2011; Armonk, NY: IBM Corp.).

## 3. Results

**Figure 3:**
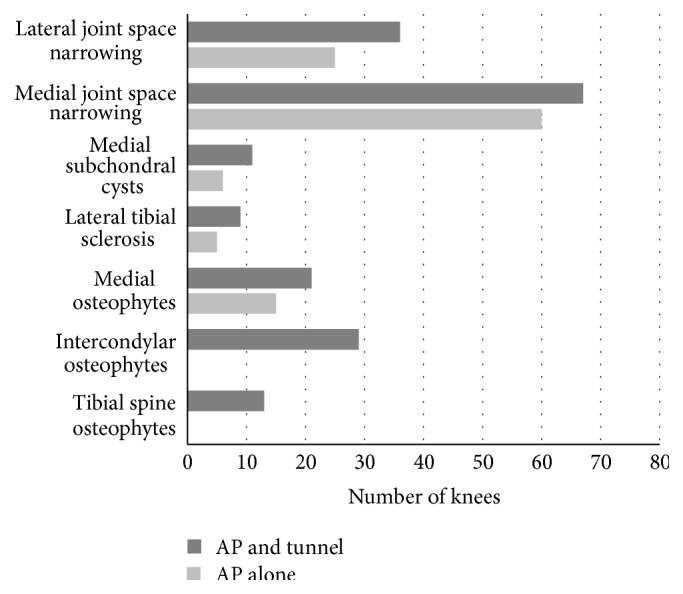
The utilization of the tunnel view significantly increased the number of knees with visible degenerative changes.

**Figure 4:**
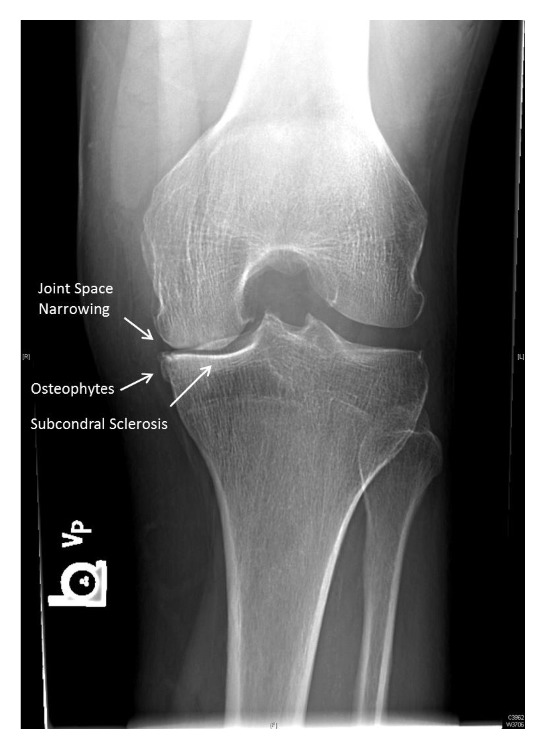
Examples of JSN, osteophytes, and subchondral sclerosis on a tunnel view radiograph.

The final analysis (Table 1) included 54 left and 46 right knees. The patients ranged from 40 to 95 years of age (mean = 68.9 years), and 64% (*n* = 48) were women. In the lateral compartment, the AP view alone detected 25 knees with JSN of at least 50%; the addition of the tunnel view significantly increased this number to 36 (*p* < 0.001). In the medial compartment, joint space narrowing of at least 50% was visible in 60 knees; using the tunnel view in conjunction significantly increased this number to 67 (*p* = 0.006). The tunnel view significantly increased the detection of subchondral cysts in the medial compartment (*p* = 0.022) and sclerosis of the lateral tibial plateau (*p* = 0.041). The use of the tunnel view also increased the detection of moderate-to-large osteophytes in the medial compartment (*p* = 0.012), the intercondylar notch (*p* < 0.001), and the tibial spine (*p* < 0.001) (Figure 3). All radiographic images, both AP and tunnel, showed at least some radiographic defects on analysis; no knees showed zero radiographic defects (Figure 4). Interrater reliability ranged from 0.72 for medial JSN and 0.84 for lateral JSN to 0.97 for lateral and femoral osteophytes. All other interrater reliability scores were within that range, which are in accordance with previously reported data [24, 25].

The tunnel view did not significantly increase the visualization of medial tibial plateau sclerosis, or medial or lateral femoral condylar sclerosis. There was no increase in the detection of loose bodies in the medial or lateral compartments, subchondral cysts in the lateral compartment, or osteophytes in the lateral compartment either. Subchondral defects on the tibial and femoral sides of both compartments did not experience an increased visualization using the tunnel view.

**Figure 5:**
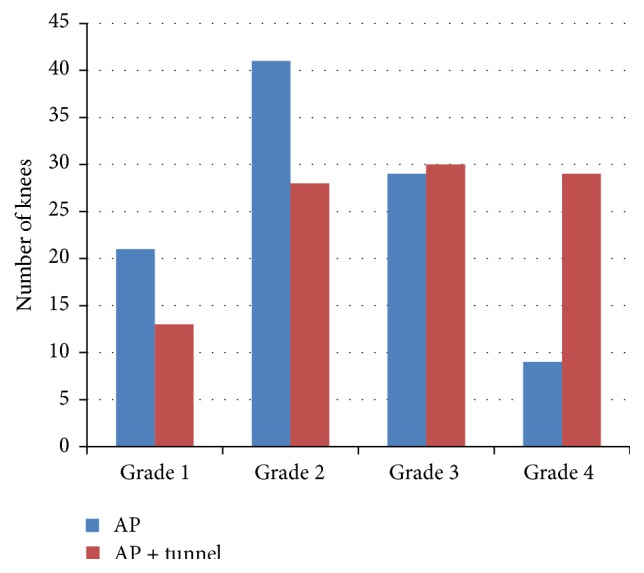
Kellgren-Lawrence scores and the number of associated knees.

Additionally, a Kellgren-Lawrence score was applied to each knee using data from our eDCF. With the addition of the tunnel view, 46 of the knees increased in KL score severity: nine knees changed from grade 1 to 2, 17 knees from 2 to 3, four knees from 2 to 4, and 16 knees from 3 to 4. KL totals and score changes can be seen in Table 2 and Figure 5.

Of the 100 knees, surgery was recommended on 56 of these knees and 35 surgeries were completed. Twenty-one total knee arthroplasties, 11 medial unicondylar knee arthroplasties, two lateral unicondylar knee arthroplasties, and one orthoscopic meniscus repair were performed. There were only two cases in which the intraoperative findings of osteoarthritis contradicted the radiographic findings and in these cases, patients with planned unicondylar knee arthroplasties underwent total knee arthroplasties. Only one unicondylar knee arthroplasty required revision to a total knee arthroplasty at a later date.

To relate this to clinical treatment options we analyzed the data by looking at shifts in compartment degenerative changes with relationship to whether one used the AP, tunnel, or both views. We found that the AP view alone detected 13 knees with bicompartmental (both medial and lateral compartments) joint space narrowing of at least 50%, 47 knees with isolated medial narrowing, and 12 knees with isolated lateral compartment narrowing. Utilizing the tunnel view in conjunction with the AP view, 25 knees showed bicompartmental joint space narrowing, 42 had isolated medial narrowing, and 11 had isolated lateral disease. This represents a shift of two knees from lateral to bicompartmental narrowing, eight knees from medial only to bicompartmental narrowing. Of the knees with no clinically significant joint space narrowing seen on the AP view, the addition of the tunnel view identified joint space narrowing in the lateral compartment in one knee, the medial compartment in three knees, and both compartments in two knees (Table 3).

## 4. Discussion

Prior studies have compared the tunnel view directly to the AP view. Rosenberg et al. analyzed the AP and tunnel view radiographs of 55 knees and found the degree of joint space narrowing visualized in the tunnel view correlated more frequently with the findings in an arthroscopic evaluation [2]. A 2007 study of 202 knees demonstrated that the Schuss view more frequently identified definitive joint space narrowing than the AP view [26]. In a review of 50 patients by eight physicians, the use of the Schuss view was demonstrated to have a significant impact on clinical decision-making [4], while a prospective analysis by Davies et al. confirmed the importance of the Schuss view compared to full extension for identifying tibiofemoral OA [27]. A 2007 evaluation of 309 knees demonstrated the tunnel view's superiority in the visualization of certain features of joint degeneration, especially within the intercondylar space; however, this study did only analyze the tunnel view with respect to anterior knee pain [16]. Additionally, the research done by Davies et al. showed the importance of the WB PA in flexion as a separate tool, rather than in combination with the standard, fully extended AP radiograph [27].

All of these studies compare the AP directly to the tunnel view, which we do not believe to be a comparison of much clinical utility. The AP is a gold standard of diagnostic imaging and should not be replaced by the tunnel view. Of more interest to our group was what the addition of the tunnel view would do for our ability to radiographically detect degenerative changes in the knee.

In this study, the tunnel views significantly aided in visualization of joint space narrowing. In contrast to data reported by Yamanaka et al. [28], our identification was most significant within the lateral compartment, where the tunnel view increased the number of knees with clinically significant narrowing by 44%. In the medial compartment, the number of knees with significant narrowing increased by 12%. A possible reason for this increase is due to a more robust visualization of the joint line in both extension and partial flexion.

Analyzing the joint space narrowing data on a per knee basis provides a demonstration of the tunnel view's possible effect on clinical decision-making. The location of joint degeneration in a knee, whether bicompartmental or isolated to the medial or lateral compartments, could be used to assist in the determination of treatment options available to the patient, both surgical and nonoperative treatments. For example, in the eight knees from our study that initially appeared to have isolated joint space narrowing in the medial compartment, a possible treatment option could have been a medial UKA; however, with the identification of joint space narrowing in the other compartments, TKA could possibly be a better treatment option. In our study, we found eight knees that initially appeared to have isolated joint space narrowing in the medial compartment, potentially candidates for medial UKA. This improved visualization of the knee through the tunnel view image was also seen in knees that initially had no narrowing or isolated lateral compartment narrowing.

Since this study reviewed a consecutive series of subjects presenting with knee pain, many of the subjects did not go on to have an operation; therefore, there is no direct clinical correlation to cartilage deterioration within our subjects. While arthroscopic confirmation of cartilage evaluation is ideal [29], previous studies have shown that joint space width and narrowing reliably measure cartilage thickness, thinning, and compression in the medial compartment and that the JSN in the lateral compartment was predictive of and comparable to the medial compartment for cartilage loss [27, 30]. While MRIs would provide a 3D assessment of the knee, as opposed to the 2D assessment provided by plain radiographs [31], by improving the visualization of the knee joint, as seen in the tunnel view image in combination with the standard AP film, extra testing and imaging may be possibly avoided. While there may be a slight increase in cost and in radiation dosage received by the patient due to the extra X-ray image, the extra benefits provided by the tunnel view image support the addition of the image to standard knee work-up.

In addition to the significant joint space narrowing changes, the addition of the tunnel view provided significant identification differences in sclerosis of the lateral tibial compartment, subchondral cysts in the medial compartment, and osteophytes in the medial compartment, intercondylar notch, and tibial spine. The improved visualization of the intercondylar notch and the tibial spine using the tunnel view can be attributed to rotation of the notch structure. One reason for the difference in identification on the tibial aspect of the knee is perhaps due to natural tibial slope, often quoted as 7°. Since the WB AP image is often taken with the knee in full extension and the beam perpendicular, visualization of the back of the tibia is hard to discern, based on the tibial slope. The tunnel view X-ray is not taken at this perpendicular angle, allowing for more visualization of the tibia. While slightly unexpected, the tunnel view did also increase the visualization of medial condylar spurs. This identification can possibly be attributed to the idea that spurs are often more visible in flexion than in extension.

The authors recognize that there are limitations to this study. In the study, we are studying radiographic osteoarthritis and not necessarily the true symptomology or gold standard for osteoarthritis. However, radiographic evaluation is often used as a standard to evaluate many patients, and in conjunction with the physical exam, shown to be very accurate. Due to the subjective nature of radiographic readings, we had three separate physicians to read each image to enhance precision. Additionally, since this was an analysis of sequential subjects presenting to the office with generic knee pain, the characteristics observed may be a self-selective group rather than the population as a whole. Despite these limitations, we believe that this study provides important information as to the utility of the WB tunnel view.

In summary, the tunnel view radiograph is an important tool that can be used in conjunction with the AP view for the evaluation of knee pain due to the ability to detect radiographic signs of osteoarthritis not seen by the AP image alone. Also, the information supplied by the tunnel view in conjunction with the AP can assist with the determination of possible treatment options provided to the patient. For these reasons, we recommend the WB tunnel view be included in the standard radiographic evaluation of any patient with knee pain.

## Figures and Tables

**Table 1:** The degenerative changes visualized in 100 knees.

Degenerative change	Compartment	AP	AP + tunnel	*p* value
Joint space narrowing	Lateral	25	36	<0.001^*∗*^
	Medial	60	67	0.006^*∗*^

Tibial sclerosis	Lateral	5	9	0.041^*∗*^
	Medial	16	19	0.079

Femoral sclerosis	Lateral	1	3	0.153
	Medial	8	8	1.000

Subchondral cysts	Lateral	3	4	0.315
	Medial	6	11	0.022^*∗*^

Loose bodies	Lateral	0	0	1.000
	Medial	2	4	0.153

Subchondral tibial defect	Lateral	1	1	1.000
	Medial	2	3	0.315

Subchondral femoral defect	Lateral	0	0	1.000
	Medial	1	1	1.000

Osteophytes	Lateral	15	17	0.153
	Medial	15	21	0.012^*∗*^
	Intercond. notch	0	29	<0.001^*∗*^
	Tibial spine	0	13	<0.001^*∗*^

^*∗*^Significant value.

**Table 2:** Kellgren-Lawrence score and associated changes with the addition of the tunnel view.

KL score	AP #	Tunnel #	KL change	KL change #
Grade 1	21	13	Grade 1 → 2	9
Grade 2	41	28	Grade 2 → 3	17
Grade 3	29	30	Grade 2 → 4	4
Grade 4	9	29	Grade 3 → 4	16
			No change	54

**Table 3:** The addition of the tunnel view shifted the compartments with detectable joint space narrowing.

#	AP alone	AP + tunnel
10 knees	Unicompartmental	Bicompartmental^*∗*^
4 knees	No arthritis	Unicompartmental
2 knees	No arthritis	Bicompartmental^*∗*^

^*∗*^Bicompartmental = both medial and lateral compartments.
